# Phenotypic characterization of paroxysmal dyskinesia in Maltese dogs

**DOI:** 10.1111/jvim.15804

**Published:** 2020-05-16

**Authors:** Dakir Polidoro, Luc Van Ham, Patrick Santens, Ine Cornelis, Marios Charalambous, Bart J. G. Broeckx, Sofie F. M. Bhatti

**Affiliations:** ^1^ Small Animal Department, Faculty of Veterinary Medicine Ghent University Merelbeke Belgium; ^2^ Department of Neurology, Faculty of Medicine and Health Sciences Ghent University Hospital Ghent Belgium; ^3^ Laboratory of Animal Genetics, Faculty of Veterinary Medicine Ghent University Merelbeke Belgium

**Keywords:** canine, involuntary movement, limb dystonia, movement disorder, seizure

## Abstract

**Background:**

Paroxysmal dyskinesias (PDs) are a group of central nervous system diseases characterized by episodes of abnormal involuntary hyperkinetic movement without altered consciousness that increasingly have been recognized in dogs.

**Objectives:**

To present the phenotypical characterization, treatment, and outcome of a PD observed in Maltese dogs.

**Animals:**

Client‐owned Maltese dogs (n = 19) with presumed diagnosis of PD.

**Methods:**

Data were collected retrospectively from medical records (2014‐2019), and supporting information was added prospectively by using a questionnaire directed to the owners of the affected dogs.

**Results:**

The episodes were characterized mainly by sudden dystonia of ≥1 limbs and generalized body tremors with preserved consciousness. The mean age of clinical onset was 5.4 years. Episode frequency varied widely both among and within individuals. Median episode duration was 4.5 minutes. Most episodes were stress‐ or exercise‐induced. Acetazolamide was administered to 6 dogs, and 4 dogs experienced a decrease in episode frequency. In 7 dogs that received a gluten‐free diet, 6 dogs became episode‐free. In 4 dogs, the episodes stopped spontaneously and in 2 dogs no medication or specific diet was given and the episodes continued at the same frequency.

**Conclusions and Clinical Importance:**

Given the breed predisposition and regional distribution of the disease, additional research should focus on elucidating the underlying genetic cause doing so might advance both our understanding of the pathophysiology and treatment of this disease, not only in dogs, but also in humans. Regardless of the treatment protocol selected, prognosis appears fair to good.

**Abbreviations**
BCAN: brevican gene
CSF: cerebrospinal fluid
EEG: electroencephalography
MRI: magnetic resonance imaging
PD: paroxysmal dyskinesia
PNKD: paroxysmal nonkinesigenic dyskinesia

## 1 INTRODUCTION

Paroxysmal dyskinesias (PDs) are a heterogeneous group of hyperkinetic and recurrent episodes of abnormal involuntary movement that are self‐limiting. Usually, autonomic signs are absent, consciousness is not impaired, and abnormal postictal behavior is not observed.^1^ Episodes can last from seconds to minutes or even hours, and the movement disturbance begins and stops abruptly. These features aid in distinguishing PDs from epileptic seizures, the most important differential diagnosis for this condition.^1^

Although PDs remain poorly characterized in the veterinary literature, they have become an increasingly recognized condition over the last decade, especially because of the fact that smartphone technology has enabled dog owners to record episodes at home and show them to their veterinarians.^2^, ^3^, ^4^, ^5^, ^6^, ^7^, ^8^, ^9^, ^10^

In human medicine, PDs can be classified clinically in 3 major categories, including paroxysmal kinesigenic dyskinesia (usually brief [<2 minutes] episodes typically precipitated by sudden movements, which can be exacerbated by stress, menses, cold, and heat), paroxysmal nonkinesigenic dyskinesia (PNKD; episodes of involuntary movements that occur spontaneously, lasting minutes to hours) and paroxysmal exertion‐induced dyskinesia (episodes induced by prolonged sustained exercise, lasting between seconds to 30 minutes).^11^ Most PDs described in dogs resemble PNKD in humans but, a recent study proposed a clinical classification in veterinary medicine, including genetic, secondary (eg, drug‐induced PD and structural intracranial disease), dietary, and unidentified (presumed genetic) causes.^1^ Until now, genetic sequencing has identified 2 causative genetic mutations related to PD in dogs: episodic falling syndrome in Cavalier King Charles Spaniels, resulting from a mutation in the brevican gene (*BCAN*)^12^, ^13^ and PD in soft‐coated Wheaten Terriers, which has been associated with a mutation in the *PIGN* gene.^10^ Paroxysmal gluten‐sensitive dyskinesia is a well‐described type of PNKD in Border Terriers, characterized by episodes of difficulty walking, tremors, and dystonia of the limbs, head, and neck, and gastrointestinal signs that can be observed between episodes and that improve when a gluten‐free diet is fed.^3^, ^8^, ^14^, ^15^ For unidentified (presumed genetic) causes, a PNKD has been described in Chinook dogs and is characterized by an inability to stand, head tremors and involuntary flexion of ≥1 limbs.^2^ In addition, “Scottie cramp,” an exercise‐, stress‐, or excitement‐induced PD in Scottish Terriers, likely also has a genetic basis.^16^, ^17^

The natural course of PDs was self‐limiting in Labrador Retrievers and Jack Russell Terriers, with 32% of the dogs undergoing spontaneous remission and 75% showing marked improvement.^18^ Several drugs have been reported to be successful in the treatment of dogs with PD, including acetazolamide,^19^fluoxetine,^20^clonazepam,^21^diazepam,^22^ and phenobarbital.^23^

To the our knowledge, only a single abstract (from our research group) has described this condition in Maltese dogs.^24^ Therefore, the aim of our retrospective study was to describe in detail the clinical features of this paroxysmal movement disturbance, including its treatment and outcome in Maltese dogs.

## 2 MATERIALS AND METHODS

Clinical cases were identified by retrospectively searching the medical records of the Small Animal Teaching Hospital at Ghent University between 20 June 2014 and 6 November 2019 for Maltese dogs that were presented for evaluation of episodes of involuntary movements without loss of consciousness and for which video footage, a detailed description of the episodes or both were available. After inclusion, owners were contacted by email or telephone or both and were invited to complete a detailed questionnaire based on previous veterinary literature on movement disorders in dogs. The questionnaire contained information about age of onset, time period of occurrence, triggers, frequency, duration, and phenotypical characterization of the episodes (Supporting Information Addendum 1). Signalment, history, clinical and neurological examination findings, CBC, serum biochemistry profile, brain magnetic resonance imaging (MRI) findings, cerebrospinal fluid (CSF) analysis, electrodiagnostic examination, and genetic testing were obtained from the medical records.

### 2.1 Statistical analysis

Statistical analysis was conducted by R version 3.5.2 (“Eggshell Igloo”). When normally distributed, data are presented as mean and SD. Otherwise, median and range are given. The relative risk and its 95% confidence interval (CI) of the Maltese dog breed relative to the general population of dogs presented at the Small Animal Teaching Hospital at Ghent University during the same time period, was calculated. Significance was set at α ≤ .05.

## 3 RESULTS

From 4428 patients that visited the neurology unit of the Small Animal Teaching Hospital at Ghent University, 19 of 125 Maltese dogs were presented for evaluation of PD (prevalence, 15.2%), whereas only 62 other cases of PD were identified in the remaining 4303 dogs (prevalence, 1.4%). This difference was significant, with a relative risk estimate for Maltese dogs relative to the general population being equal to 9.4 (95% CI, 5.7‐15.7; *P* < .001).

Fifteen dogs were male (9 neutered) and 4 were female (3 neutered). The reported age of clinical onset ranged from 1 to 11 years (mean, 5.4 years). None of the dogs had a pedigree or any recorded ancestry or lineage. In 15 dogs (79%), the episodes could be witnessed and analyzed using a video recording provided by the owners, and in 4 dogs (21%), a detailed description of the episodes was available. Most of the dogs (14) were receiving commercial dog food at the time of clinical onset, 4 dogs were receiving a hypoallergenic diet (not gluten‐free) and 1 dog was receiving natural homemade food. In 7 dogs (37%), concomitant health issues were present at the time the episodes started. One dog had signs of inflammatory bowel disease, 2 dogs had sporadic episodes of vomiting and borborygmi, 3 dogs suffered from skin allergy, and 1 dog had intermittent episodes of diarrhea and skin allergy. Of the 4 dogs with skin problems, 1 was receiving dexamethasone (Rapidexon, Dechra, Shrewsbury, UK) at the time the episodes started, and another dog was receiving oclacitinib maleate (Apoquel, Zoetis, Parsippany, New Jersey), both as treatment for the skin allergy. There was no known family history of PD in any of the affected Maltese dogs.

### 3.1 Occurrence of episodes

The episodes occurred randomly (after stress or exercise or both or at rest) in 8 dogs (42%). In 5 dogs (26%), the episodes only occurred after stress or exercise or both and, in 6 dogs (32%), only during rest or sleep. The frequency of occurrence of the episodes was variable, among and within dogs. Episodes could occur daily in the same dog, resembling clusters, with some quiescence of weeks or months and other dogs would experience larger intervals between episodes without an evident pattern. The episodes could occur at any time during the day in 16 dogs (84%), only in the morning in 2 dogs (10%) and only in the evening in 1 dog. Most dogs only had 1 episode per day (11 dogs; 58%), but 3 dogs (16%) could have up to 2 episodes per day, 1 dog up to 3 per day and in 4 dogs (21%) up to 4 times per day.

### 3.2 Characterization of the episodes

Video S1 illustrates the typical clinical features of an episode. The episode starts immediately after the dog seeks the owner's attention. The dog seems restless while generalized body tremors are evident, and starts showing dystonia in the left thoracic limb, which progressively involves all 4 limbs after adopting sternal or lateral recumbency. The dog remains conscious during the entire episode. The clinical signs disappear gradually and spontaneously over the course of approximately 4 minutes.

Most of the owners were not able to predict the occurrence of the episodes (10 dogs; 53%), but some dogs would seek their owners' attention (8 dogs; 42%) or were fearful (1 dog) immediately before an episode. All owners reported that their dogs were conscious and aware during the episodes, being able to respond when called by their name, although the episodes could not be stopped when the dog was distracted by touching or calling its name. All owners described dystonia‐like disturbances (stiffness and cramping of ≥1 muscle groups) in at least 1 of the limbs during the episodes (only pelvic limbs in 6 dogs [32%], all limbs in 7 dogs [37%], only thoracic limbs in 3 dogs [16%], or randomly 1 limb in 3 dogs [16%]). All owners described generalized body tremors (including trunk and head) and difficulty in walking when attempted by the dog. In 9 dogs (48%), a lateral body posture was adopted during the episodes, whereas 5 dogs (26%) adopted a sternal position and the other 5 (26%) were in standing position. A kyphotic posture was seen in 3 dogs (16%). Only 5 dogs (26%) adopted salivation during the episodes, of which 2 (10%) also had single episodes of vomiting. All dogs were clinically and neurologically normal before and between the episodes, but 3 dogs (16%) showed a short period (<2 minutes) of fatigue and panting after the episode occurred, and 1 dog was disoriented for approximately 30 minutes. The episodes lasted 1‐5 minutes (10 dogs; 53%), 5‐10 minutes (4 dogs; 21%), 10‐30 minutes (3 dogs; 16%), or >30 minutes (2 dogs; 10%). Overall, episodes ranged from 1‐90 minutes, with a median time of 4.5 minutes.

### 3.3 Diagnostic evaluation

General physical and neurological examinations, CBC, and serum biochemistry profile were unremarkable in all 19 dogs. Magnetic resonance imaging of the brain and CSF analysis were performed in 2 dogs (10%), and electrodiagnostic examination (electromyography, motor nerve conduction velocity and repetitive nerve stimulation) were performed in 1 dog (5%) and all were unremarkable. Three dogs (16%) tested negative for the *PIGN* mutation.^10^

### 3.4 Clinical course, treatment, and outcome

Acetazolamide at a dosage of 4 mg/kg PO q12h or q8h was initiated in 6 dogs (32%). The episodes decreased in frequency in 4 dogs (21%), but none of the dogs became episode‐free. In 2 dogs (10%) treated with acetazolamide, no improvement was seen and 1 of them experienced severe adverse effects, including apathy and drowsiness. Acetazolamide was discontinued in both dogs and they were treated with fluoxetine at a dosage of 2 mg/kg PO q24h. One dog became episode‐free after treatment with fluoxetine, and the other dog's episode frequency did not change and it was presented for evaluation of loss of appetite, possibly caused by the medication. This dog recently was given a gluten‐free diet, but no improvement was seen. Dietary changes (gluten‐free diet) were made in 7 dogs (37%), and 6 of them became episode‐free after instituting the new diet. In 1 dog that received a gluten‐free diet, episode frequency remained the same but the duration of individual episodes decreased. In 6 dogs (32%), no diet or medication was given. In 4 of these dogs, the episodes stopped spontaneously and in 2 the episodes continued at the same frequency.

Of the 7 dogs that had concomitant health issues at the time the episodes started, only 1 was treated with a gluten‐free diet. This patient was showing episodes of vomiting and borborygmi, which resolved after the new diet was started as primary treatment for the PD.

## 4 DISCUSSION

At the Small Animal Teaching Hospital at Ghent University, paroxysmal movement disorders have been diagnosed clinically in various breeds, but our results seem to indicate that the Maltese breed is over‐represented. We are not aware of any other reported cases other than those reported by our group. The high number of Maltese dogs with PD in our region might suggest a genetic or familial component, although this remains to be proven. Unfortunately, none of the Maltese dogs in our study had pedigrees, which precluded any pedigree analysis. Our aim was to describe the clinical features of a PD in Maltese dogs and to describe its clinical course, treatment, and outcome.

Most dogs previously reported with PD have a mean age of onset of <1 year,^4^, ^19^, ^25^, ^26^, ^27^, ^28^ but it could vary from 2 to 4 years in a few reports.^5^, ^7^, ^8^, ^9^, ^14^, ^18^, ^29^, ^30^ On the contrary, the mean age of the first experienced episode in our Maltese dogs was higher (mean, 5.4 years; range, 1‐11 years).

Paroxysmal dyskinesia affecting Maltese dogs resembles PNKD in humans, where the episodes are defined as attacks of involuntary movements that occur spontaneously.^11^ In 42% of our dogs, the episodes occurred randomly (after stress or exercise or both or at rest). In 26% of the cases, the episodes started only after stress or exercise. Stress (42%) and exercise (23%) also have been associated as a trigger in a group of Norwich Terriers.^7^ Contrary to dyskinesias observed in a group of Border Terriers in which the episodes occurred mainly at rest for the majority of the dogs (87%),^30^ in our study the episodes occurred during rest or sleep only in 6 Maltese dogs (32%). Two Maltese dogs experienced an episode during the neurological examination, possibly as a result of stress. In other dogs with PD, owners could predict an episode because their dogs would seek their attention before an episode,^3^, ^9^ as was seen in 8 dogs (42%) in our study. As described in humans with PNKD, patients are able to report an aura‐like sensation immediately before an episode, characterized by limb stiffness or paresthesia or an undefined feeling.^31^ Dogs might have a similar sensation, which could explain why they would seek the attention of the owner before an episode occurs. As reported in other breeds with PD, all affected Maltese dogs in our study remained conscious during the episodes, which could not be stopped even when the dog was stimulated by physical or vocal prompting by the owners. Although the episodes could not be interrupted, the preservation of consciousness during the generalized episodes, despite motor manifestations in all 4 limbs, is supportive of a diagnosis of PD. For this reason, a movement disorder would be considered more likely than a generalized tonic‐clonic seizure.^1^, ^3^ Paroxysmal dyskinesias also are differentiated from focal epileptic seizures by a lack of seizure activity on ictal electroencephalography (EEG), which is the ideal method to distinguish both conditions.^2^, ^11^ None of the Maltese dogs in our study underwent EEG during an episode because only 2 dogs had unexpected episodes during consultation. Interictal EEG might have been useful in our dogs, because we would expect no epileptic seizure activity on EEG. In humans, interictal scalp EEG is used routinely to record the electrical signals of the brain and is considered an effective method for defining the epileptogenic zone^32^ in approximately 70% of patients with epileptic seizure activity.^33^ In veterinary medicine, ictal or interictal EEG presents logistical and technical limitations,^2^, ^7^ because of the low likelihood of witnessing an event^3^ and limited availability of equipment.^27^ Even when feasible, interpretation of ictal EEG might be challenging in dogs presenting with PD because muscle activity may cause artifacts.^6^

Five Maltese dogs (26%) presented with salivation, vomiting, or both during the episodes. Although affected animals do not usually exhibit autonomic dysfunction,^1^, ^4^ a recent study reported abnormal autonomic activity during episodes of PD in Border Terriers in 56% of the cases, most commonly hypersalivation and vomiting in 48 and 24% of the affected dogs, respectively.^30^ Furthermore, autonomic signs also have been reported in other studies.^6^, ^9^ In 2 previous reports,^2^, ^3^ dogs presumably diagnosed with PD that showed autonomic signs were excluded from the study. Consequently, autonomic abnormalities might be more common than originally thought during PD events. Presence of autonomic signs has been reported to be a factor that can be used to distinguish PDs from epileptic seizures,^2^, ^4^, ^29^ but based on what was observed in our study and described in the veterinary literature, this distinction might not be helpful anymore. Furthermore, we cannot necessarily conclude that these clinical signs in the 5 Maltese dogs are abnormal autonomic signs and not a consequence of a possible multisystem disorder, as seen in PD in Border Terriers.^8^

In our study, the episodes mainly were characterized by sudden dystonia (sustained muscle contraction producing abnormal movements and postures) affecting ≥1 limbs, the trunk and the head. Such clinical signs have been reported in other breeds, including Norwich Terriers,^7^ Bichon Frises,^29^ Yorkshire Terriers,^27^ Golden Retrievers,^19^ Border Terriers,^3^ Chinook dogs,^2^ Jack Russel Terriers, and Labrador Retrievers.^18^ In general, dogs diagnosed with PD tend to have an episode duration <5 minutes,^7^, ^18^, ^28^, ^30^ which was the case in 10 Maltese dogs (53%) in our study.

The underlying mechanism of PD remains unknown, and its diagnosis is speculative in many cases.^7^, ^18^ Similar to dogs with idiopathic epilepsy, dogs with PD are clinically and neurologically normal between episodes, and thus structural intracranial pathology seems unlikely.^3^, ^27^, ^29^, ^30^ Clinicians should take this likelihood into account when choosing the most cost efficient diagnostic approach.

Initially, patients diagnosed with PD were treated with acetazolamide, based on evidence in the current veterinary literature.^19^ Recently, the benefits of a gluten‐free diet in dogs with PD have become more established.^9^, ^14^ Acetazolamide also might have beneficial effects as was observed in a previous report.^19^ The majority of the dogs in our study treated with acetazolamide experienced a decrease in episode frequency. Acetazolamide is a carbonic anhydrase inhibitor that results in kaliuresis, diuresis, and metabolic acidosis and also lowers serum bicarbonate concentrations, which decreases the amount of brain lactate and pyruvate, resulting in brain acidosis and changes in intracellular and extracellular pH.^34^ The change in pH in the brain parenchyma alters the membrane potential, culminating in a decreased neuronal excitability.^35^ The suppressive effect on neuronal excitability is the main mechanism of action in humans with PD.^36^ Six dogs that were fed a gluten‐free diet became episode‐free. The benefits of a gluten‐free diet already have been observed in other breeds with PD.^3^, ^7^, ^9^, ^26^ Some of the Maltese dogs in our study were presented with signs of gastrointestinal disease, skin allergy, or both. These signs also were seen in Border Terriers diagnosed with gluten‐sensitivePD,^8^, ^14^, ^30^ in which serological markers of gluten sensitivity (anti‐transglutaminase‐2 and anti‐gliadin antibodies) were detected in affected dogs. These titers decreased after a gluten‐free diet was initiated. Because the majority of our Maltese dogs that received a gluten‐free diet responded well to dietary change, future research should focus on a possible relation between serological markers of gluten sensitivity and Maltese dogs with PD. Also, the effect of a gluten‐free diet on concomitant signs such as gastrointestinal disease or skin problems warrants further investigation. Spontaneous remission occurred in 21% of the affected Maltese dogs (n = 4), a similar percentage compared to other breeds previously described.^18^ Although most dogs seem to respond positively to the different types of treatments offered, it is important to emphasize the possible natural course of this condition and the so‐called placebo phenomenon reported for epilepsy drug treatment trials.^37^ It has been observed previously that episodes have a tendency to stabilize in the first few years after the beginning of clinical signs, at which point they may improve in most cases and resolve completely in a few cases.^18^ Therefore, the natural course of PD in untreated dogs should be considered before attributing remission to specific treatment effects.^18^

Our study had some limitations. A complete diagnostic evaluation was not performed in all patients, including electrodiagnostic studies (EEG), MRI, and CSF analysis. In addition, a detailed pedigree or recorded ancestry together with further genetic investigation could have added evidence to our conclusions. The retrospective character of our study, use of data obtained from a questionnaire and the fact that most of the episodes were identified through video footage also were limitations. To investigate the effect of dietary or medical treatment, a double‐blinded randomized placebo‐controlled trial would have been more appropriate before drawing definitive conclusions.

The finding of a paroxysmal movement disorder with characteristics specific for a single breed is highly suggestive for a genetic predisposition. Future research should focus on elucidating the genetic basis of PD in Maltese dogs because doing so may promote understanding of the pathophysiology and improve treatment.

## 5 CONCLUSION

A PD, which resembles PNKD in humans, has been characterized phenotypically in Maltese dogs. Given the breed predisposition and regional distribution of the disease, a genetic basis is likely. Overall, disease progression is variable and, regardless the treatment used, prognosis appears fair to good. Interestingly, several cases in our study population experienced spontaneous clinical remission without any treatment.

## CONFLICT OF INTEREST DECLARATION

Authors declare no conflict of interest.

## OFF‐LABEL ANTIMICROBIAL DECLARATION

Authors declare no off‐label use of antimicrobials.

## INSTITUTIONAL ANIMAL CARE AND USE COMMITTEE (IACUC) OR OTHER APPROVAL

Authors declare no IACUC or other approval was needed.

## HUMAN ETHICS APPROVAL

Authors declare human ethics approval was not needed for this study.

## Supporting information


**Data S1** Addendum 1: Questionnaire.

**Video S1** A Maltese dog experiencing the typical clinical features of an episode of paroxysmal dyskinesia. Note the generalized body tremors and the initial dystonia in the left thoracic limb which progressively involves all 4 limbs. The dog remains conscious during the entire episode.
