# Supplementary material for: Phenotypic characterization of paroxysmal dyskinesia in Maltese dogs
Source: J Vet Intern Med. 2020 May 16;34(4):1541–6. doi: 10.1111/jvim.15804 (PMC7379016; doi:10.1111/jvim.15804)
Supplement: Supplementary file 1 — Data S1 Addendum 1: Questionnaire. [file JVIM-34-1541-s001.pdf]

Addendum 1: Questionnaire

Sex:

Male □ Female □

Date of birth:………………...

Pedigree:

Yes □ No □

Video footage of the episode:

Yes □ No □

Diet the dog is currently taking (specify type and brand):

Was the dog suffering from any other condition while the episodes started?

□ Hypersensitivity (allergic) skin condition □ Gastrointestinal problems

□ Traumatic event □ Other:………………………

Was the dog on any medication while the episodes started:

Yes □ (specify) No □

Is there any family history with the same symptoms:

Yes □ No □

Episode's details

1) Age of onset

a) Dog's age at which you first observed an episode:

b) Date of last known episode:

2) Occurrence of episodes

a) Is there any trigger able to elicit the episodes:

□ Stress/excitation □ Happens during rest/sleep

□ Exercises □ Noise □ Other:

b) Which time of the day are the episodes more frequent:

□ Morning (6am-12am) □ Afternoon (12am-5pm) □ Evening (5pm-8pm)

□ Night (8pm-6am) □ Random

c) What is the frequency of the episodes:

□ Daily □ Weekly □ Monthly □ Other (describe):

d) In case of daily episodes, what is the frequency:

□ 1 time □ 2 times □ 3 times □ 4 times □ Random

3) Characterization of the episodes

a) Is it possible to predict the episodes?

□ Yes □ No

b) Does the dog appear conscious during the episode:

□ Yes □ No

c) Does the dog appear to be aware of surroundings during the episode:

□ Yes □ No

d) Does the dog attempt to come to you during the episode:

□ Yes □ No

e) Can you get the dogs' attention during the episode (calling/touching):

□ Yes □ No

f) Does the dog exhibit trouble walking during the episode:

□ Yes (describe) □ No

g) Does the dog tend to lie down during the episode:

□Yes (□ lateral □ sternal) □ No (standing)

h) Does the dog show unusual head/body movements during the episode:

□ Yes (describe) □ No

i) Does the dog show salivation/defecation/urination during the episode:

□ Yes (describe) □ No □ Other (describe):

j) Is the dog normal after/before the episodes:

□ Yes □ No

k) Is the dog normal between the episodes:

□ Ye □ No

l) Which limbs are mainly affected during the episodes:

□ Both thoracic limbs □ Both pelvic limbs □ All limbs

□ Other (describe):

m) What is the average length of an episode:

□ <5 mins □ 5-10 mins □ 10-30 mins □ >30 mins (describe):
